# Promoting Caregiver Early Childhood Development Behaviors through Social and Behavioral Change Communication Program in Tanzania

**DOI:** 10.3390/ijerph19095149

**Published:** 2022-04-23

**Authors:** Eliza Broadbent, McKayla McConkie, Emily Aleson, Lily Kim, Rebekah Stewart, Generose Mulokozi, Kirk A. Dearden, Mary Linehan, Dennis Cherian, Scott Torres, Ben T. Crookston, Parley C. Hall, Joshua H. West

**Affiliations:** 1 Department of Public Health, Brigham Young University, Provo, UT 84602, USA; elizabroadbent@gmail.com (E.B.); mckaylamarin@gmail.com (M.M.); emilysearle95@gmail.com (E.A.); raheekim1029@gmail.com (L.K.); bekah.stewart5@gmail.com (R.S.); benjamin.crookston@gmail.com (B.T.C.); coughall@gmail.com (P.C.H.); 2 IMA World Health Tanzania, Dar es Saalam 14111, Tanzania; generose.mulokozi@gmail.com; 3 Corus International, Washington, DC 20036, USA; kdearden@corusinternational.org (K.A.D.); marylinehan609@gmail.com (M.L.); dcherian@corusinternational.org (D.C.); 4 RTI International, Washington, DC 20005, USA; torscott@gmail.com

**Keywords:** early childhood development, mass media, interpersonal communication

## Abstract

**Objectives:** Stunting remains a prevalent issue in Tanzania. The consequences of stunting include reduced height, greater susceptibility to disease, and diminished cognitive ability throughout the lifespan. Lack of psychosocial stimulation is associated with increased stunting, particularly in terms of its cognitive impact. The Addressing Stunting in Tanzania Early (ASTUTE) program was a large social and behavior change communication (SBCC) intervention that aimed to reduce childhood stunting in the region by targeting early childhood development (ECD) behaviors. The purpose of this study is to report on the extent to which exposure to ASTUTE might be related to ECD behaviors. **Methods:** ASTUTE disseminated program messages via a mass media campaign and interpersonal communication (IPC). Logistic regression models were used to explore the relationship between exposure to TV, radio, IPC messages, and key ECD behaviors of female primary caregivers and male heads of household. **Results:** Among primary caregivers, IPC was positively associated with all ECD behaviors measured. Radio was associated with all behaviors except drawing with their child. TV was associated with all behaviors except playing with their child. Among heads of household, only the radio was positively associated with the ECD behaviors measured. **Conclusions for practice:** Findings indicate that SBCC interventions that include mass media and IPC components may be effective at promoting parental engagement in ECD behaviors. **Significance:** We know that ECD is important for a child’s development. We know that parents play a critical role in promoting ECD behaviors. We are still exploring ways to influence parents so that they are more involved in ECD behaviors. The results presented here provide evidence for successful mass media and IPC efforts to improve parents’ ECD behaviors. We hope this study will add more evidence for large interventions such as these to the literature, and we are very hopeful that governments and large international NGOs will prioritize SBCC approaches in the future, especially in locations where face-to-face interventions may be challenging.

## 1. Introduction

Approximately 150 million children under the age of five suffer from stunting globally [1]. Over two million children, mostly living in South Asia and sub-Saharan Africa, fail to reach their developmental potential because of stunting [2]. The World Health Organization defines stunting as having a height-for-age more than two standard deviations below average [3]. Stunting can lead to severe infections, reduced psychosocial stimulation, lower educational outcomes, and shorter stature in adulthood [4]. Developmental impairments caused by stunting reduce economic potential in later life, as stunted adults may have more difficulty finding jobs and earn lower wages [4]. Further research indicates that stunting impacts early childhood development (ECD), decreasing cognitive ability and fine/gross motor skills [4,5,6].

A variety of factors contribute to the persistence of stunting in developing nations such as Tanzania. Food security is a substantial challenge to proper child nutrition [2]. It is a persistent problem in Tanzania, where many individuals cannot consistently access a sufficient food supply to satisfy their needs for a healthy life [7]. This is particularly damaging in the first 1000 days of life (from conception to age two). Children who are poorly nourished during this period are likely to fail to reach their full growth and developmental potential [6]. Beyond food security, cultural factors such as gender inequality may also play a role [8]. Given Tanzanian households’ patriarchal structure, male heads of household often make health decisions independently of their female partners [9]. This structure frequently results in women and children eating cereal, vegetables, seeds, and nuts to the exclusion of critical animal source foods such as beef, poultry, fish, milk, or eggs [10]. In addition to cultural practices, other psychosocial factors impact stunting. These include lower levels of maternal and paternal education, where a lack of knowledge about childcare, breastfeeding, and hygiene [11] and a lack of awareness and ability to provide psychosocial stimulation during early childhood often result in child undernutrition [5].

ECD and early childhood experiences can impact physiological [12] and neural developments [13] and can impact stunting independent of nutrition [14,15]. Childhood stimulation is a key component of fostering healthy ECD. A study performed on paternal engagement in stimulation highlighted the importance of having at least one engaged caregiver present to provide stimulating opportunities to the child [16]. Another study in rural Peru focused on teaching mothers methods on how to stimulate and interact with their young children at home via playing with toys, talking and interacting with the child, and creating a safe environment for child play. The intervention improved ECD across many domains, such as the relationship between objects, communication, and motor skills [17]. Pitchik et al. [18] investigated ECD in relation to parental involvement in Tanzania. Children who experienced higher levels of parental stimulation scored better in areas of cognition/language. Greater stimulation and higher maternal education also improved reported child motor scores. It is likely that integrated interventions pertaining to the environment, education, and caregiver-child stimulation can impact childhood development outcomes in resource-limited settings such as Tanzania [18].

The purpose of this study was to explore the association between exposure to an SBCC intervention using mass media and interpersonal communication (IPC), targeting childhood stunting and maternal and paternal behaviors related to ECD in a region of Tanzania. Given the strength of the literature on IPC interventions and psychosocial factors [19,20], we postulated that IPC exposure would be strongly associated with improved female caregiver behaviors regarding ECD. We predicted that a similar, although weaker, association would be found for exposure to the mass media campaign given the larger, more generic nature of such interventions [21,22,23]. Even less is known about the influence of media interventions on the behavior of the male head of the household; thus, it was predicted that a weak relationship would be present.

## 2. Methods

Addressing Stunting in Tanzania Early (ASTUTE) involved a large SBCC nutrition project that included mass media communications and IPC interventions focused on improving children’s nutrition and development indicators before a child reaches the age of 2 in order to decrease stunting. IMA World Health led the implementation of this program with funding from UK Aid and the Foreign, Commonwealth & Development Office (FCDO). ECD was a key theme of the program. The theory-based radio spots were broadcast a total of 70,000 times, and each ended with a consistent tagline. TV spots were aired before and during the evening news on national and regional stations for a total of 1198 times. The IPC component of the intervention was implemented by community health workers (CHW) during 30-minute in-home visits. The CHWs counseled mothers an average of 3.6 times in-home and referred children with faltering growth to health facilities for treatment while also educating and supporting mothers to engage in stimulation-related behaviors such as drawing, playing, naming objects, and talking with children. More details about the mass media communications and IPC interventions can be found elsewhere [24].

### 2.1. Sample

A total of 4996 mothers, hereafter known as primary caregivers, and 3082 corresponding fathers, hereafter known as male heads of the household, were surveyed across the Lake Zone region of Tanzania, which includes the five regions of Geita, Kagera, Kigoma, Mwanza, and Shinyanga. Only households with a child under the age of 2 years were eligible to participate. A stratified, multi-stage random sample design was used to select questionnaire participants. Within the five participating regions, 243 villages were selected, and participants were randomly sampled within each village. Participant demographics are presented in Table 1.

### 2.2. Study Design and Procedure

The questionnaire was administered to participants following the mass media campaign. Questionnaire items were directed at the female caregiver of the youngest child in the home and the male head of household if available and applicable. The research firm IPSOS collected data and used a field team that consisted of 10 supervisors and 50 enumerators. In total, 25% of records were quality-checked by conducting revisits and phone checks. IRB approval was granted by Development Media International’s (DMI) internal IRB and Tanzania’s National Institute for Medical Research. Participation was voluntary, and informed consent was collected before the survey began. Respondents were told that they could stop the survey at any time. Questionnaire items were written in English and then translated into Swahili. The Swahili items were then checked by DMI and IPSOS to ensure that original intent was retained. The questionnaire was piloted and adjusted before being given to participants. The questionnaire contained 169 questions and required approximately 50–60 min to complete.

### 2.3. Measures

Data were collected on participant demographic characteristics, reported exposure to the intervention, and engagement in key ECD practices.

**Wealth.** Household wealth was estimated using a calculated composite variable comprising multiple questionnaire items that were adapted from a previously validated index [25]. The index comprised two sub-indices, including access to services and ownership of consumer durables. Access to services pertains to the availability of safe drinking water sources (e.g., protected wells and a public standpipe) and safe sanitation (e.g., a flush toilet). For this study, pit latrines were not counted as safe sanitation. Consumer durables included ownership of seven items: a radio, TV, bicycle, motorcycle, mobile phone, boat, or animal-drawn cart. An average of the two indices was used to calculate an overall wealth score, with possible values ranging between 0 and 1. Higher wealth scores indicate higher socioeconomic status.

**SBCC Intervention Exposure.** A separate exposure score was calculated for each of the radio, TV, and IPC intervention components. Exposure to radio was coded “yes” if respondents reported yes to hearing the example spots that endwith a laughing baby sound, or if they reported hearing messages on the radio that advised about maternal/child health/child developments. Exposure to TV was coded “yes” if respondents reported yes to seeing the example image frame(s) on TV or “reported seeing messages on the TV that advised about maternal/child health/child development.” IPC exposure was coded “yes” if respondents reported that they had received an in-home visit from a (community) health worker that advised about maternal/child health/child developments. Exposure to radio, TV, and IPC was estimated for female primary caregivers. IPC targeted females only, so male respondents responded only to questions about exposure to radio and TV and not IPC.

**ECD Behaviors.** Eight items were used to measure female primary caregivers’ ECD behaviors. Four items pertained to talking to children. Examples included “In the last week, have you talked to [your child] about other people in your family?” and “In the past week, have you spoken to [your child] even if they were unable to respond?” Given their thematic similarity, items that pertained to talking were combined into a cumulative talking measure with scores ranging from 0 (answered “no” to all four talking items) to 4 (answered “yes” to all four talking items). The remaining items asked primary caregivers about their practices related to drawing (“In the last week, have you done any drawing with [your child]?”), counting (“In the last week, have you counted things in front of [your child]?”), naming objects (“In the last week, when you have been with [your child], have you named the objects around you so that your baby starts learning words?”), and playing (“In the last week, have you spent time playing with [your child]? (e.g., chasing, playing a game, playing with a toy)”) with their child. Item responses were dichotomized, with the “don’t know” response option coded as missing. Two items were used to measure male heads of households’ ECD behaviors: “In the last week, have you spent time playing with [your child]? (e.g., chasing, playing a game, playing with a toy)” and “When you are holding [your child], do you name the objects around you? (e.g., cow, house, sister, tree).” Item responses were dichotomized, with the “don’t know” response option coded as missing.

### 2.4. Analysis

To ensure that data remained confidential, they were deidentified and shared only with study personnel. The dataset with survey results was cleaned and re-coded in STATA version 16. Further data analysis was performed in SAS 9.4. Frequency statistics were calculated for key demographic variables, intervention exposures, and ECD indicators. Logistic regression was conducted for the ECD variables with each of the exposure variables. All models were adjusted for respondent age, education, and household wealth. Each model was examined for goodness-of-fit and each met acceptable standards.

## 3. Results

The average age of female primary caregivers was 28.23 years. The majority (56.87%) had completed primary school, although nearly one-third reported education levels less than a primary education level. Almost 87% were married, with divorced and never married being the next most common categories. More than half reported a pension, NGO aid, or a family member as their largest income source, although a substantial portion (40.15%) reported self-employment as their primary source. The sample mostly (85.99%) reported living in an urban setting. On average, the male heads of household were older than primary caregivers, with a mean age of 34.55 years. Most (64.28%) reported primary school as their highest education level, with 18.56% receiving at least some secondary schooling. The most common main source of income was self-employment. The average wealth score for households included in the sample was 0.36 (RANGE: 0–1). Table 1 contains the full results for demographic variables.

Reported exposure to intervention messages via radio, TV, and IPC channels is displayed in Table 2. For both primary caregivers and heads of household, the radio was the most common media exposure. More than 56% of primary caregivers reported hearing intervention messages via radio spots, compared to 23.52% via TV. A higher percentage of heads of household (38.28%) had seen intervention messages on TV compared to female primary caregivers. However, radio exposure was still more prevalent (65.92%) than TV among heads of household. Only female primary caregivers were exposed to IPC, and the fewest respondents reported exposure to this component of the intervention (see Table 2).

Most female primary caregivers reported engaging in three of the five ECD behaviors measured. Nearly three-fourths (74.76%) practiced all four talking behaviors in interactions with their child, while a slightly higher percentage played with or named objects for them: 75.08% and 77.22%, respectively. Heads of household had similarly high participation in the “playing” and “naming” ECD practices (see Table 3). The prevalence of ECD behaviors of “drawing” and “counting” was much lower, with only a fourth of primary caregivers having drawn with their child and 38.55% having counted with their child.

Table 4 presents the adjusted regression analysis results assessing the relationship between exposure to intervention messages and the ECD indicators for primary caregivers. Reported exposure to radio was associated with talking (OR = 1.41; CI 1.23–1.61), counting (OR = 1.35; CI 1.2–1.52), naming (OR = 1.35; CI 1.18–1.55), and playing (OR = 1.19; CI 1.04–1.36). Reported exposure to TV was associated with talking (OR = 1.3; CI 1.1–1.53), drawing (OR = 1.35; CI 1.15–1.58), counting (OR = 1.47; CI 1.27–1.69), and naming (OR = 1.26; CI 1.06–1.51). Furthermore, the relationship between IPC exposure and increased engagement in ECD was significant for all indicators, including talking (OR = 1.66; CI 1.39–1.99), drawing (OR = 1.68; CI 1.44–1.96), counting (OR = 1.60; CI 1.39–1.85), naming (OR = 1.36; CI 1.14–1.63), and playing (OR = 1.24; CI 1.04–1.46).

Table 5 presents the results of the adjusted regression analysis of intervention exposure and the ECD indicators for heads of household. Only radio exposure was associated with increased odds of reporting the two ECD behaviors measured. Male heads of household who heard radio spots with intervention messages were 1.42 times more likely to name objects for their child (OR = 1.42; CI 1.19–1.7) and 1.58 times more likely to play with their child (OR = 1.58; CI 1.31–1.92) compared to those that reported no exposure to the radio messages.

## 4. Discussion

The purpose of this study was to determine if exposure to an SBCC intervention that included IPC and mass media was related to female primary caregiver and/or male head of household ECD behaviors. As hypothesized, IPC exposure was associated with more ECD indicators than radio or TV exposure. The relationship between male head of household exposure to media and ECD behaviors was mixed. Radio exposure, but not TV, was associated with ECD behaviors. This may have been because the total number of radio spots was more than 50-times greater than the TV spots. Nevertheless, all significant associations were in the expected direction such that greater exposure was related to an increased likelihood of engagement in ECD behaviors.

### 4.1. Impact on Female Primary Caregivers

IPC was associated with improved maternal ECD behaviors across all indicators. These findings from Tanzania support previous reports that IPC interventions can improve child development [17,26]. It has also been found that IPC interventions are feasible, viewed as acceptable by participants [27], and are effective in influencing maternal behaviors [28].

Exposure to mass media messages, including television and radio channels, was also related to ECD behaviors in the current study. Other studies have similarly found mass media campaigns to be an effective means of promoting behavioral change, if appropriately planned and executed [29,30,31]. A meta-analysis of mass media interventions concluded that well-targeted interventions to a population could be successful in changing both behaviors and attitudes [31]. Another review reported a dose–response effect in that a more extended intervention period and greater intervention intensity/exposure were related to increased effectiveness [29]. The mass media intervention implemented in Tanzania may have proved successful due to its extensive reach in the population and messages tailored to the population.

Although the IPC component in this study was strongly related to female caregivers’ ECD behaviors, it reached a much smaller proportion of the population than the other interventions. While this study does not compare the magnitude of the impact of the various intervention components, considering the reach of each approach is important. For example, it has been noted that an intervention with the ability to reach a greater audience, even with a small-to-moderate effect (such as mass media interventions), may induce a more significant impact when compared to a more robust intervention that reaches fewer individuals [31]. These findings imply that mass media campaigns in areas such as Tanzania may have a larger impact in positively influencing ECD behaviors. Future studies could explore the extent to which each intervention component reaches a unique audience and how unique exposures relate to ECD behaviors.

### 4.2. Impact on Male Heads of Household

Most reports of the effects of mass media on child health outcomes in developing settings focus on mothers. Less is known about the impacts mass media interventions have on the behaviors of fathers. The current study indicates a positive association between radio exposure and the male head of household ECD behaviors. Those exposed to radio intervention were more likely to play with their children and more likely to name the objects around them when holding their child compared to those who were not exposed. This finding is important, especially in Tanzania, where the father’s role is still traditionally defined as the breadwinner, with fewer expectations for engagement in their child’s wellbeing and development [32,33]. The greater exposure to radio in Tanzania may be linked back to the 1990s, when widespread radio distribution was led by large donors in the country [34]. In 2018, it was estimated that 81% of farmers in a rural area of Tanzania had access to a radio, compared to only 21.6% of farmers having access to a television [35]. Additionally, because of the gender-based divisions of work in Tanzania, the mother’s household duties often take her away from home, leaving the male at home with the ability to listen to the radio [35]. Even while at home, a mother’s workload may be too demanding to allow them time to listen to the radio. These findings imply that radio interventions may be more impactful on fathers in a country like Tanzania because of increased radio access.

### 4.3. Limitations

The findings from this study should be interpreted in the context of three prominent limitations. This study was cross-sectional, making it difficult to attribute effects on ECD behaviors to the specific intervention components. Controlling for age, education, and household wealth increases confidence in the observed relationships, but it is still possible that other factors influenced the observed association. Another limitation relates to the potential for self-reporting bias. Individuals may have answered in a certain way due to cultural norms or perceived expectations relating to respondents’ perceptions of what the researchers wanted to hear. There is no reason to believe that these biases would differ depending on the reported exposure to the various intervention components. Finally, there were more primary caregiver than male head of household respondents. It is not clear how this impacted the findings, but it is important to note the disparity. There were no statistical comparisons between primary caregivers and male heads of household.

## 5. Conclusions

A strength of this study was the presence of both mass media and an IPC component for the SBCC. The additional use of radio and TV spots resulted in a far greater population reach, since a relatively small proportion could participate in IPC intervention. These results also suggest that although IPC interventions are effective, populations with access to a radio or TV may also benefit from the inclusion of mass media campaigns in interventions. Overall, our study findings indicate that SBCC, inclusive of both mass media and IPC interventions, induced a positive impact by improving ECD behaviors in both mothers and fathers. These interventions have the potential to reduce stunting in resource-poor settings.

## Figures and Tables

**Table 1:** Participant Demographics.

Parent	Variable	*N* (%)/Mean (SD)
Female Primary Caregiver		
	Age	28.23 (7.08)
	Wealth	0.36 (0.17)
	Education	
	Less than primary	1481 (29.64)
	Complete primary	2841 (56.87)
	Some secondary or more	674 (13.49)
	Sources of Income *	
	Pension, NGO aid, or family member	2759 (55.22)
	Outside employment	166 (3.32)
	Self-employed	2006 (40.15)
	No source of income	65 (1.30)
	Marriage Status	
	Never married	286 (5.73)
	Married	4340 (86.96)
	Widowed	62 (1.24)
	Divorced	15 (0.30)
	Separated	288 (5.77)
	Number of children under five	1.66 (0.80)
	Setting	
	Rural	4296 (85.99)
	Urban	700 (14.01)
	Region	
	Geita	826 (16.53)
	Kagera	1171 (23.44)
	Kigoma	959 (19.20)
	Mwanza	1312 (26.26)
	Shinyanga	728 (14.57)
Male Head of Household		
	Age	34.55 (8.49)
	Wealth	0.36 (0.17)
	Education	
	Less than primary	529 (17.16)
	Completed primary	1981 (64.28)
	Some secondary or more	572 (18.56)
	Source of Income	
	Pension, NGO aid, or family member	58 (1.88)
	Outside employment	320 (10.38)
	Self-employed	2698 (87.54)
	No source of income	6 (0.19)

* Categories are not mutually exclusive.

**Table 2:** Participants Exposed to Radio, TV, and IPC Interventions.

Parent	Intervention	*N* (%)
Primary Caregiver		
	Radio	2822 (56.49)
	TV	1175 (23.52)
	IPC	980 (19.62)
Head of Household		
	Radio	2014 (65.92)
	TV	1154 (38.28)

**Table 3:** Participants Reporting Practice of Key Early Childhood Development Behaviors.

Parent	Key Indicators	*N* (%)
Primary Caregiver		
	Talking	
	No talking behaviors	45 (0.90)
	One talking behavior	82 (1.64)
	Two talking behaviors	127 (2.54)
	Three talking behaviors	1007 (20.16)
	All four talking behaviors	3735 (74.76)
	Drawing	1225 (24.53)
	Counting	1926 (38.55)
	Naming	3858 (77.22)
	Playing	3751 (75.08)
Head of Household		
	Naming	2335 (75.81)
	Playing	2493 (80.92)

**Table 4:** Regression Analysis for Primary Caregiver’s Exposure to Radio, TV, and IPC Intervention and Key Practice Indicators, *n* = 4996.

Key Indicators	Radio		TV		IPC	
	Odds Ratio (CI)	*p*-Value	Odds Ratio (CI)	*p*-Value	Odds Ratio (CI)	*p*-Value
Talking	1.41 (1.23–1.61)	<0.0001 ***	1.3 (1.1–1.53)	0.002 **	1.66 (1.39–1.99)	<0.0001 ***
Drawing	1.06 (0.93–1.22)	0.385	1.35 (1.15–1.58)	0.000 **	1.68 (1.44–1.96)	<0.0001 ***
Counting	1.35 (1.2–1.52)	<0.0001 **	1.47 (1.27–1.69)	<0.0001 **	1.60 (1.39–1.85)	<0.0001 ***
Naming	1.35 (1.18–1.55)	<0.0001 **	1.26 (1.06–1.51)	0.009 **	1.36 (1.14–1.63)	0.001 **
Playing	1.19 (1.04–1.36)	0.011 *	1.08 (0.92–1.27)	0.358	1.24 (1.04–1.46)	0.014 *

Note: Each model controlled for mother’s age, mother’s level of education, and household wealth. * *p* < 0.05, ** *p* < 0.01, *** *p* < 0.001.

**Table 5:** Regression Analysis for Male Heads of Household Exposure to Radio, TV, and IPC Intervention and Key Practice Indicators, *n* = 3082.

Key Indicators	Radio		TV	
	Odds Ratio (CI)	*p*-Value	Odds Ratio (CI)	*p*-Value
Naming	1.42 (1.19–1.7)	<0.0001 ***	1.19 (0.99–1.42)	0.068
Playing	1.58 (1.31–1.92)	<0.0001 ***	1.2 (0.98–1.46)	0.075

Note: Each model controlled for father’s age, father’s level of education, and household wealth. *** *p* < 0.001.

## Data Availability

Data can be made available upon request.
